# GATAD2B regulates spindle assembly by affecting protein deacetylation during oocyte meiotic maturation

**DOI:** 10.5713/ab.25.0013

**Published:** 2025-06-04

**Authors:** Qian Xu, Lina Yu, Yuling Lin, Aolei Guo, Yang Zhang, Zhe Zhang, Guijun Yan, Haixiang Sun, Guangyi Cao

**Affiliations:** 1 State Key Laboratory of Reproductive Medicine and Offspring Health, Center for Reproductive Medicine and Obstetrics and Gynecology, Nanjing Drum Tower Hospital Clinical College of Nanjing Medical University, Nanjing, China; 2 Center for Reproductive Medicine and Obstetrics and Gynecology, Nanjing Drum Tower Hospital, Affiliated Hospital of Medical School, Nanjing University, Nanjing, China; 3 Jiangsu Human Reproductive Function Remodeling Engineering Research Center, Nanjing, China

**Keywords:** Deacetylation, GATAD2B, Meiosis, Oocyte, Spindle

## Abstract

**Objective:**

Oocyte quality is critical for the stable transmission of genetic information and affects early embryonic development. But the precise mechanisms governing oocyte meiotic progression remains largely unclear. Transcription regulation through chromatin compaction and decompaction is regulated through various chromatin-remodeling complexes such as nucleosome remodeling and histone deacetylation (NuRD) complex. GATAD2B is known to be a component of the NuRD complex but whether GATAD2B regulates chromatin modification in mouse oocyte meiosis remains unclear. We hope to explore the role of GATAD2B in mouse oocyte meiosis.

**Methods:**

In this study, we initially utilized western blot and immunofluorescence to delineate the expression and subcellular localization of GATAD2B during oocyte meiotic maturation. To further elucidate the role of GATAD2B in regulating oocyte meiotic division, we employed the method of microinjection of *Gatad2b*-specific siRNA to knock down the protein expression of GATAD2B. Subsequently, dynamic changes in oocyte meiotic division were captured in real-time using live-cell imaging with Geri. Additionally, spindle staining, DNA staining, spread analysis, and reanalysis of RNA-seq data were performed to investigate the mechanisms through which GATAD2B regulates oocyte meiotic division.

**Results:**

GATAD2B was stably expressed during oocyte meiosis and was significantly increased during the metaphase II (MII) stage. To further explore the effect of GATAD2B on oocyte meiotic maturation, we observed increased abnormal spindle, severe chromosome misalignment and metaphase I (MI) block in GATAD2B knocked-down (GATAD2B-KD) oocytes. Interestingly, the distribution of microtubule organizing center was abnormal and aneuploidy was significantly increased in GATAD2B-KD oocytes. In addition, some deacetylation-related genes were significantly downregulated and acetylated proteins accumulated abnormally in GATAD2B-KD oocytes.

**Conclusion:**

These findings implicate GATAD2B as a novel regulator of histone deacetylation during oocyte maturation and provide evidence that such deacetylation is required for proper spindle assembly.

## INTRODUCTION

Female fertility decreases with age, and one of the main manifestations of this is the increase in aneuploidy due to abnormal spindle assembly and chromosome segregation during oocyte maturation [1,2]. The process of oocyte senescence is accompanied by dynamic changes in histone acetylation, in which insufficient histone deacetylation leads to increased aneuploidy rates in aged female mice [3,4]. In old germinal vesicle (GV) oocytes, acetylation of histone H4K12 and H4K16 decreased [4]. The acetylation levels of histone H3K14, H4K8 and H4K12 in mouse oocytes gradually increased during aging *in vivo* and *in vitro* after ovulation [3]. In contrast, inhibition of histone deacetylases (HDACs) leads to abnormal spindle assembly during oocyte maturation [5,6]. These results suggest that histone acetylation plays an important role during oocyte maturation.

Acetylation and deacetylation of histones are catalyzed by histone acetyltransferases (HATs) and HDACs, respectively. Different histone acetylation patterns are recognition codes that recruit different transcription factors upon transcriptional activation [7]. There is a dynamic balance between the activities of HAT and HDAC. Upon resumption of meiosis, oocytes undergo a reduction in histone acetylation associated with maturation [8,9]. The gene expression pattern of differentiated oocytes is reprogrammed during meiosis to allow the next generation of allotropic zygotes to initiate a new program [8]. Inhibition of HDAC activity (HDAC11, HDAC3, etc.) during oocyte maturation leads to histone hyperacetylation, which in turn alters meiotic progression [5,6,10]. Although HDACs affect gene expression during oocyte maturation by controlling histone acetylation, it is not clear how HDACs are regulated during oocyte maturation.

Nucleosome remodeling and histone deacetylation (NuRD) complex is a 1 MDa multi-subunit protein complex which comprises many different subunits, including *Hdac1/2*, *Mta1/2*, *Rbbp4/7, Chd3/4*, and *Gatad2a/2b* [11,12]. Among them *Gatad2b*, also called p66b, recruits *Mbd2*, another component of the NuRD complex, to DNA and histones, affecting histone deacetylation and repression of transcription [13]. An opposing regulation of *Gatad2b* by *Mkk6* phosphorylation elevates histone acetylation levels and enhances pluripotency gene expression [14]. Recently, *Gatad2b* was reported to be sumoylated to enhance the formation of NuRD complexes [15,16]. And *Gatad2b* can directly interact with histone acetylation sites H3k9ac, H3K27ac and H4K16ac [14].

*Rbbp7* is one of the components of the NuRD complex, a dormant maternal mRNA that is recruited for translation during oocyte maturation to regulate histone deacetylation. During oocyte meiotic maturation, RBBP7 loss of function leads to abnormalities in chromosomal passenger complex (CPC) localization and function [17]. RBBP4 is a ubiquitously expressed nuclear protein that belongs to the WD-40 family [18]. In mouse oocytes, RBBP4 can regulate bipolar spindle assembly by promoting Aurora kinase (AURK) C function [19]. Deletion of RBBP4 and RBBP7 function both result in oocyte histones H3K4, H4K8, H4K12, and H4K16 exhibit acetylation hyperacetylated, increasing the incidence of abnormal spindles, chromosome misalignment, and aneuploidy at metaphase II (MII) [17,19], and this deacetylation is required for bipolar spindle assembly via AURKC [19].

In this article, we demonstrated that *Gatad2b* is a novel regulator of histone deacetylation during meiosis maturation of mouse oocytes. Depletion of *Gatad2b* leads to abnormal spindle formation during metaphase I (MI) meiosis, followed by first polar body extrusion defects, chromosomal misalignment, and increased aneuploidy of MII. Intriguingly, we examined that the localization and activity of the microtubule organizing center (MTOC) complex was impaired within *Gatad2b*-depleted oocytes. Our contributions reveal new insights into the regulation of *Gatad2b* function and its role, especially associated with histone deacetylation, in spindle assembly.

## MATERIALS AND METHODS

### Animals

All animal experiments were conducted according to the guidelines of the Institutional Animal Care and Use Committee of Nanjing Drum Tower Hospital (2025AE01013). ICR mice were purchased from Beijing Vital River Laboratory Animal Technology. Mice were fed regular chow and housed in a controlled room under a 12:12-hour light-dark cycle at 22°C.

### Oocyte collection and culture

Fully grown oocytes at GV-stage were achieved from the ovaries of 3 weeks old females. The female mice were superovulated with 5 IU pregnant mare serum gonadotropin, PMSG (Prospec) by intraperitoneal injection 46–48 hours ago. Female mice were used for oocyte retrieval 46–48 hours after intraperitoneal injection of 5 IU of PMSG (Sansheng). Cumulus-oocyte complexes were collected and cumulus cells were removed by mouth pipetting repeatedly. Oocytes was collected in MEM-alpha containing 3 mg/mL of bovine serum albumin (BSA, Sigma-Aldrich) and 5 μmol/L Milrinone (Calbiochem) to prevent meiotic resumption. Oocytes medium for *in vitro* maturation was MEM (Gibco, USA) supplemented with 3 mg/mL BSA, 75 μg/mL penicillin G (Sigma-Aldrich), 50 μg/mL streptomycin sulfate (Sigma-Aldrich), 25 μg/mL pyruvate (Sigma-Aldrich) and 38 μg/mL EDTA (Sigma-Aldrich). The oocytes were obtained under mineral oil at 37°C in a 5% O_2_, 5%CO_2_ and 90%N_2_ incubator.

### Antibodies

Rabbit polyclonal antibodies were purchased from proteintech: anti-GATAD2B (Cat#:25679-1-AP, 1:200) and anti-γ-TUBB (Cat#:15176-1-AP, 1:400). Rabbit polyclonal antibodies were purchased from abcam: anti-H4K16ac (Cat#: ab109463, 1:400). Mouse monoclonal FITC conjugated anti α-tubulin (Cat#: F2168; 1:500), was purchased from Sigma-Aldrich. Human antibody, anti-centromere CREST (Cat#: 15-234; 1:500), was purchased from Antibodies Incorporated. 555-Rhodamine (TRITC) donkey anti-human IgG (Cat#:709-025-149; 1:750) was purchased from Jackson Immuno-Research Laboratory. Mouse anti-β-actin (Cat#:AF0003; 1:1,000) was purchased from Beyotime. Rhodamine-Phalloidin (Cat#: YP0063-50T; 1:1,000) was purchased from US EVERBRIGHT. Alexa Fluor Plus 555 donkey anti-rabbit IgG (Cat#: A31572; 1:1,000) and Alexa Fluor Plus 488 donkey anti-mouse IgG (Cat#: A-21202; 1:1,000) were from Thermo Fisher Scientific. Anti-acetylcysteine (Cat#: PTM-101, 1:200) was purchased from PTM BIO. The specificity of the antibodies was confirmed by Western blotting, showing a distinct band at the expected molecular weight.

### Microinjection

The mouse oocytes were microinjected with approximately 10 pl of siRNA (25 μM) to knock down *Gatad2b* using a FemtoJet microinjector (Eppendorf). A combination of three different siRNAs targeting *Gatad2b* (5′-GCAGCCAAUAGCG AGUUUATT-3′, 5′-GGGACAACAAGGCUUAUCUTT-3′, 5′-CCCGAUCCAUGCUUUCAAATT-3′) was purchased, diluted with RNase-free water and stored at −80°C. The control oocytes were injected with control siRNAs (GenePharma). After injection, the oocytes were cultured in the medium containing 5 μM milrinone for 24 h to maintain GV-arrest. The oocytes were then cultured in fresh medium under mineral oil at 37°C in a 5% O_2_, 5%CO_2_ and 90%N_2_ incubator.

### Immunofluorescence

Mouse oocytes were fixed in 4% paraformaldehyde (PFA) at room temperature for 30 minutes and then blocked with PBS containing 5% BSA and 0.5% Triton X-100 at room temperature for 1 hour. After blocking, the oocytes were incubated with primary antibodies at 4°C overnight. Secondary antibodies were incubated at room temperature for 1 hour. The details of antibodies were provided above. The nuclei were stained with DAPI (Hoechst 33342) for 10 minutes. The oocytes were mounted on glass slides and examined under a laser scanning confocal microscope (LSM 780, Carl Zeiss). Fluorescence intensity was quantitatively analyzed by Leica software. To analyze the length of spindle poles, the measurement tools that come with Leica Software was used. All fluorescent images were taken at the same scale. One side of the spindle pole was selected using the line segment tool to the other side and the software will provide the length information. The length information was collected for analysis.

### Western blotting

A total of eighty mouse oocytes were lysed in 50 mM Tris-HCl (pH 6.8) containing 1% SDS, 1% β-mercaptoethanol, 20% glycerol, and denatured at 95°C for 5 minutes. Denatured proteins separated by 7.5% SDS-PAGE and transferred to PVDF membrane. Membranes were blocked by incubation in PBS with 5% low-fat dry milk and 0.1% Tween-20 (PBST) at room temperature for 1 hour. Membranes were then incubated with the primary antibodies, rabbit anti-GATAD2B (1:2,000, Proteintech) and mouse anti–β-actin (1:1,000, Beyotime), at 4°C overnight. Subsequently, membranes were incubated with secondary antibodies labeled with horseradish peroxidase in blocking buffer for 1 hour after washing 3 times in PBST. Finally, the signal was detected by ECL Plus Western Blotting Detection System (GE Healthcare) after washing in PBST for three times. ImageJ software was used to analyze the gray value of the obtained image, and the relative content of the target protein was calculated by the gray ratio between the target protein and the beta-actin. Finally, SPSS software was used for quantitative statistical analysis.

### Chromosome spreading

Chromosome spread was conducted as described previously [20,21]. Zona pellucida was removed with exposure to Tyrode’s buffer (pH 2.5) (T1788, Sigma-Aldrich) at 37°C for 30 seconds. The oocytes, recovering in fresh medium for ten minutes, were fixed in 1% PFA with 0.15% Triton X-100 on a glass slide. The oocytes, after air drying, were incubated with the human anti-centromere antibodies (1:500, Antibodies Incorporated) at 4°C overnight, followed by incubation with secondary antibodies, 555-Rhodamine (TRITC) donkey anti-human IgG, at room temperature for 2 h. The chromosomes were then stained with Hochest33342. The slides were examined with laser scanning confocal microscope (LSM 780, Carl Zeiss).

### Quantitative reverse transcription polymerase chain reaction analyses

Fifty oocytes for average at the indicated development with certain treatment were collected. To characterize the varied expression of different genes, total RNA was extracted, purified, reverse transcribed and amplified with Single Cell Sequence Specific Amplification Kit (P621, Vazyme) following the manufacturer’s protocol. Then, 2X Realtime polymerase chain reaction (PCR) Mix (Mei5Biotech, MF013) and Applied Biosystems Stepone Plus Real-Time PCR System (Thermo Fisher Scientific) were used to quantify the cDNA. Gene expression was normalized to GAPDH.

### Statistical analysis

Data, unless otherwise indicated, was presented as mean± standard error of the mean (SEM). Student’s t-test was used to evaluate differences between 2 groups. Multiple comparisons between more than 2 groups were analyzed by one-way ANOVA followed by Tukey’s honest significant difference (HSD) test using Prism 5.0. The differences of p≤0.05 were considered to be significant. Data are expressed as mean± SEM from at least three independent experiments.

## RESULTS

### Expression and subcellular distribution of GATAD2B during mouse oocyte meiosis

**Figure 1:**
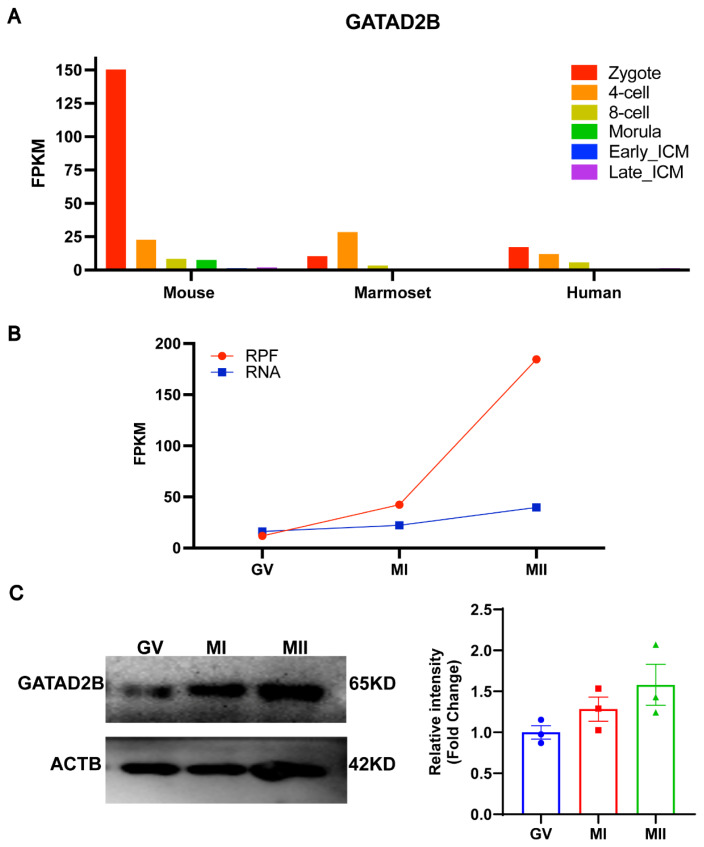
Stable expression of GATAD2B in mouse oocyte during meiotic progression. (A) Single-cell RNA-sequencing (scRNA-seq) transcriptome showed *Gatad2b* mRNA dynamics from zygote to blastocyst in mouse, marmoset and human. (B) *Gatad2b* mRNA and ribosome protected fragment (RPF) dynamics from GV to MII in mouse oocyte. (C) Western blot showed that GATAD2B protein was stably expressed during mouse oocyte meiosis. At least 80 oocytes were analyzed in each independent experiment. GV, germinal vesicle; MI, metaphase I; MII, metaphase II.

**Figure 2:**
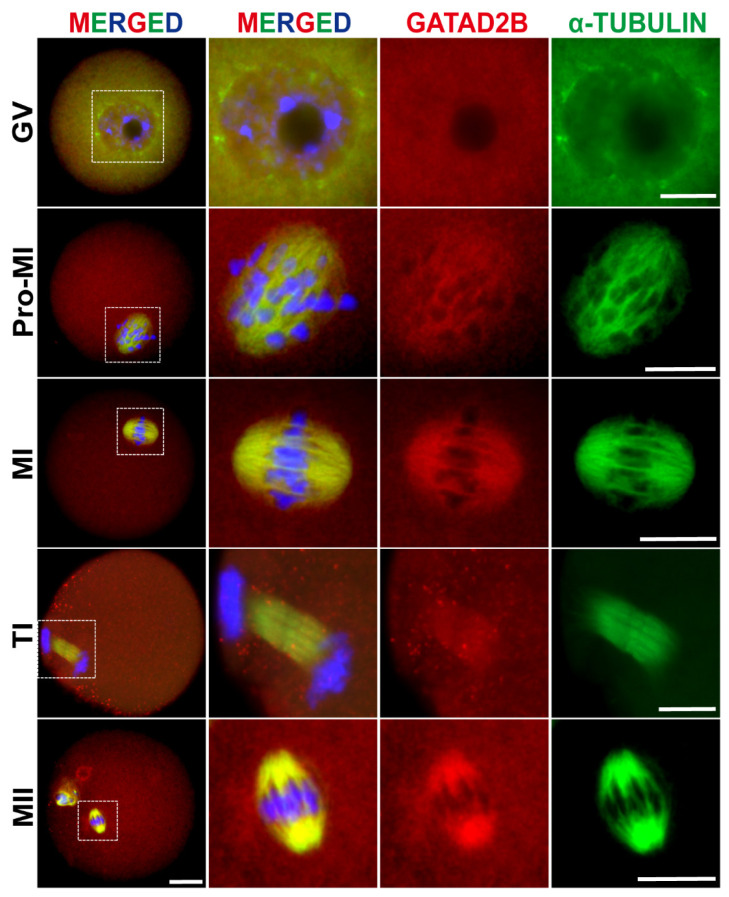
Specific localization of GATAD2B in mouse oocyte during meiotic progression. At least 10 oocytes were analyzed in each independent experiment. Scale bar, 20 μm. Red, GATAD2B. Green, α-Tubulin. Blue, DNA. DAPI was used for DNA staining. GV, germinal vesicle; pro-MI, pro-metaphase I; MI, metaphase I; TI, telophase I; MII, metaphase II.

By re-analyzing single-cell transcriptome sequencing data during human, marmoset, and mouse pre-implantation embryonic development, we found that *Gatad2b* is expressed in oocytes of three species. *Gatad2b* was expressed at abundant levels during the oocyte period, especially in mouse oocyte, while mRNA expression levels gradually decreased after fertilization (Figure 1A) [22]. To further characterize the dynamics of *Gatad2b* during oocyte meiotic maturation, we reanalyzed the mRNA level (blue line) and ribosome profiling (low-input Ribo-seq) (red line) in public database and it revealed an increase in both mRNA level and translation efficiency of *Gatad2b* from GV to MII period (Figure 1B) [23]. This result was further confirmed by western blot (Figure 1C). Subsequently, we investigated subcellular localization of GATAD2B during meiotic maturation. In GV-oocytes, GATAD2B does not exhibit specific localization. During the resumption of meiosis in oocytes, GATAD2B shows a certain degree of co-localization with α-TUBULIN at the Pro-MI and MI stages. In the TI and MII stages, GATAD2B continues to localize on the spindle (Figure 2). The specific localization of GATAD2B during oocyte maturation suggests a potential relationship between GATAD2B and spindle formation, and its involvement in regulating the process of oocyte meiotic division.

### Knockdown of GATAD2B disrupts maturational progression of mouse oocytes

**Figure 3:**
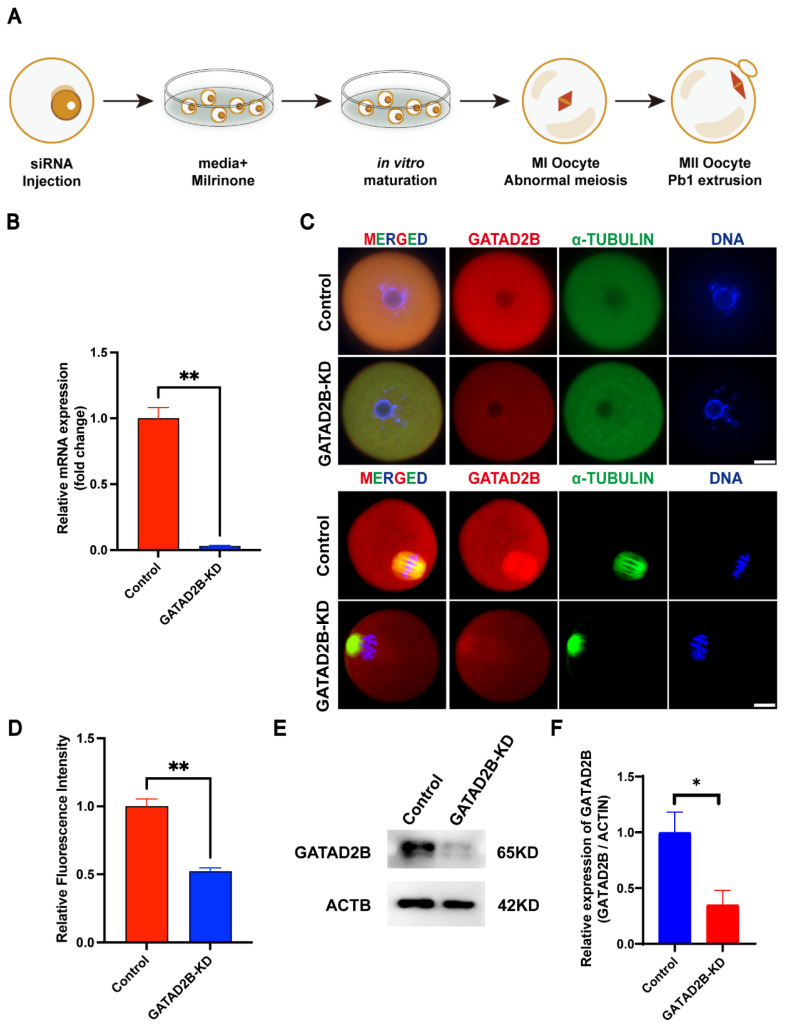
Knockdown of GATAD2B in oocytes affects oocyte meiotic maturation. (A) Impact pattern of GATAD2B-KD oocytes assessed. (B) Q-RT-PCR showing that GATAD2B was significantly knocked down by more than 90%. (C) Staining of GATAD2B oocytes shows reduced GATAD2B expression in GATAD2B-KD oocytes (GV stage and anaphase I stage). (D) The relative staining intensity of GATAD2B assessed by densitometry shows reduced GATAD2B expression in GATAD2B-KD oocytes. At least 30 oocytes were analyzed in each independent experiment. (E) Western blot analysis demonstrated efficient knockdown of GATAD2B protein levels. (F) Bar graph analysis of grayscale values displayed the knockdown efficiency of GATAD2B in oocytes. * ρ<0.05, **ρ<0.01. MI, metaphase I; MII, metaphase II; Q-RT-PCR, quantitative reverse transcription polymerase chain reaction; GV, germinal vesicle.

**Figure 4:**
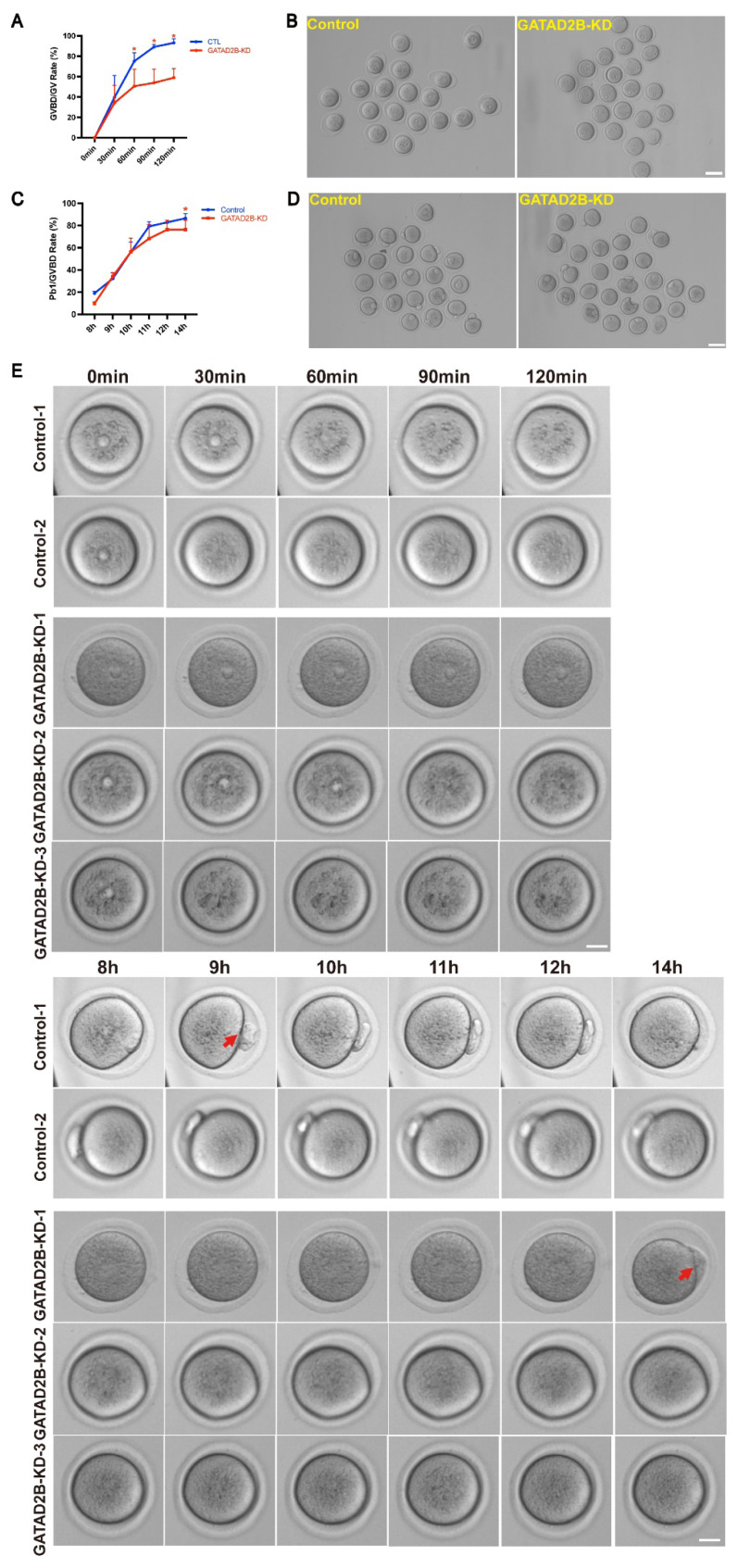
Knockdown of GATAD2B in oocytes disrupted oocyte meiotic progression. (A) GVBD rate was scored in a half-hour interval and the rate was calculated. * ρ<0.05. (B) The representative pictures of GATAD2B-KD oocytes matured *in vitro* for 1 hours are shown. (C) The first polar body (Pb1) extrusion rate of control and GATAD2B-KD oocytes was counted. Data are presented as mean±SEM. * ρ<0.05. (D) The representative pictures of GATAD2B-KD oocytes matured *in vitro* for 14 hours are shown. Scale bar, 100 μm. (E) Real-time imaging of live cells to demonstrate oocyte maturation process. The arrow indicates the first polar body. Scale bar, 20 μm. At least 30 oocytes were analyzed in each independent experiment. GVBD, germinal vesicle breakdown; GV, germinal vesicle; SEM, standard error of the mean.

In order to explore the role of GATAD2B in oocyte maturation, we reduced *GATAD2B* expression by siRNA interference in GV stage (Figure 3A). After microinjection, the GV oocytes were cultured in medium supplemented with milrinone for 20 h to facilitate *GATAD2B* mRNA degradation. The mRNA and protein expression level of GATAD2B in GATAD2B knocked-down (GATAD2B-KD) oocytes were significantly reduced (Figures 3B–3F). The germinal vesicle breakdown (GVBD) rate of GATAD2B-KD oocytes was significantly lower than that of the control group one hour after meiosis recovery. Moreover, two hours after meiosis recovery, the GVBD rate of GATAD2B-KD oocytes was still lower than 60% (Figures 4A, 4B). In addition, after eight hours of maturation *in vitro*, the extrusion of first polar body by GATAD2B-KD oocytes was delayed, compared with that by control oocytes. Even after 14 hours of maturation, the rate of Pb1 extrusion in GATAD2B-KD oocytes was notably lower than that in control group (Figures 4C, 4D). In order to observe oocyte maturation process in real time, we further confirmed the result above by live cell imaging. Indeed, it took nearly 14 hours for certain GATAD2B-KD oocytes to discharge Pb1 *in vitro* (Figure 4E). These results indicated that GATAD2B knockdown disrupted the oocyte meiosis maturation process.

### GATAD2B knockdown perturbs chromosome alignment and increases the incidence of aneuploidy in oocyte meiosis

**Figure 5:**
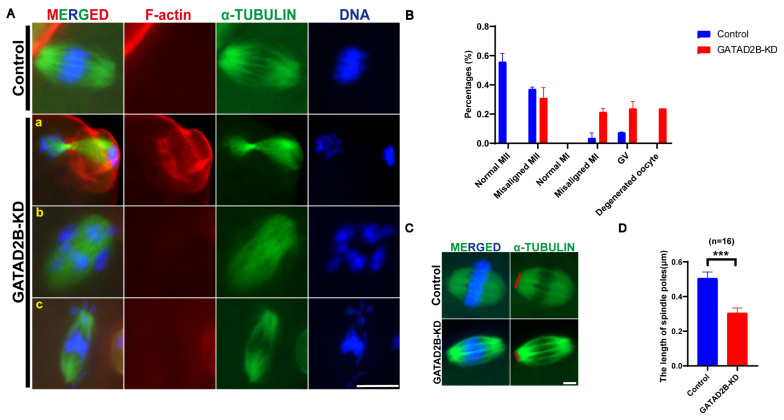
Abnormal spindle assembly due to GATAD2B knockdown. (A) Representative pictures of abnormal oocytes after GATAD2B knockdown. a, TI blocked oocyte; b, Abnormal chromosome arrangement; c, Chromosome distribution at both ends of spindle poles. Scale bar, 20 μm. (B) Bar graph showing the quantitative analysis of developmental stages (GV, Pro-MI, MI, Misaligned MI, MII, Misaligned MII, etc.) during oocyte maturation. Data are presented as mean±SEM. (C) Pattern diagram showing narrowing of spindle poles in GATAD2B-KD oocytes. Scale bar, 5 μm. (D) Statistical analysis of spindle poles length. Data are presented as mean±SEM. p = 0.0007. At least 30 oocytes were analyzed in each independent experiment. *** ρ<0.001. MII, metaphase II; MI, metaphase I; GV, germinal vesicle; SEM, standard error of the mean.

The meiosis process of oocytes was disrupted after GATAD2B knockdown, which prompted us to explore whether there were disorders in spindle assembly and chromosome arrangement. After fourteen hours *in vitro*, it was found that GATAD2B-KD oocytes, with α-Tubulin-labeled microtubules and DAPI-labeled chromosomes, had a lower normal MII-oocytes ratio (Figures 5A, 5B). The abnormal GATAD2B-KD MII-oocytes are mainly manifested as incomplete separation from the first polar body (a), disordered chromosomes (b) and spindle with abnormal shape and size (c). In the GATAD2B-KD oocytes, we looked closely at the details of spindle assembly abnormalities and found a significant narrowing of the width of the spindle poles. Spindle poles were shortened by nearly 40% in GATAD2B-KD oocytes (Figures 5C, 5D).

**Figure 6:**
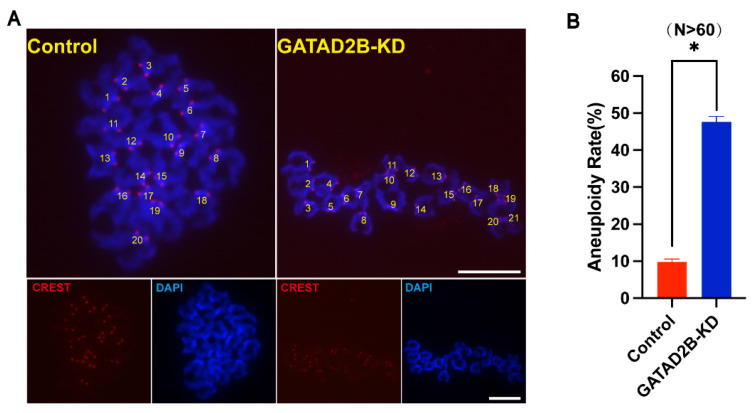
Knockdown of GATAD2B in oocytes causes abnormal increase of aneuploidy. (A) Chromosome spreading of control and GATAD2B-KD MII oocytes. Kinetochore and DNA were stained with CREST (Red) and Hoechst 33,342 (Blue), respectively. Scale bar, 5 μm. (B) The rate of aneuploidy was quantified. Data are presented as mean±SEM. * ρ<0.05. At least 30 oocytes were analyzed in each independent experiment. MII, metaphase II; SEM, standard error of the mean.

The GATAD2B-KD oocytes showed intense chromosome misalignment. Accordingly, the incidence of aneuploidy may increase eventually. To demonstrate this hypothesis, karyotype analysis of MII oocytes was performed by chromosome spreading with labeled-kinetochore. As expected, the rate of aneuploidy increased significantly in GATAD2B-KD oocytes (Figure 6). Taken together, these results suggest that GATAD2B deletion disrupts spindle assembly and chromosome alignment and leads to an increasing incidence of aneuploidy.

### GATAD2B knockdown results in abnormal distribution of microtubule organizing center in oocyte

**Figure 7:**
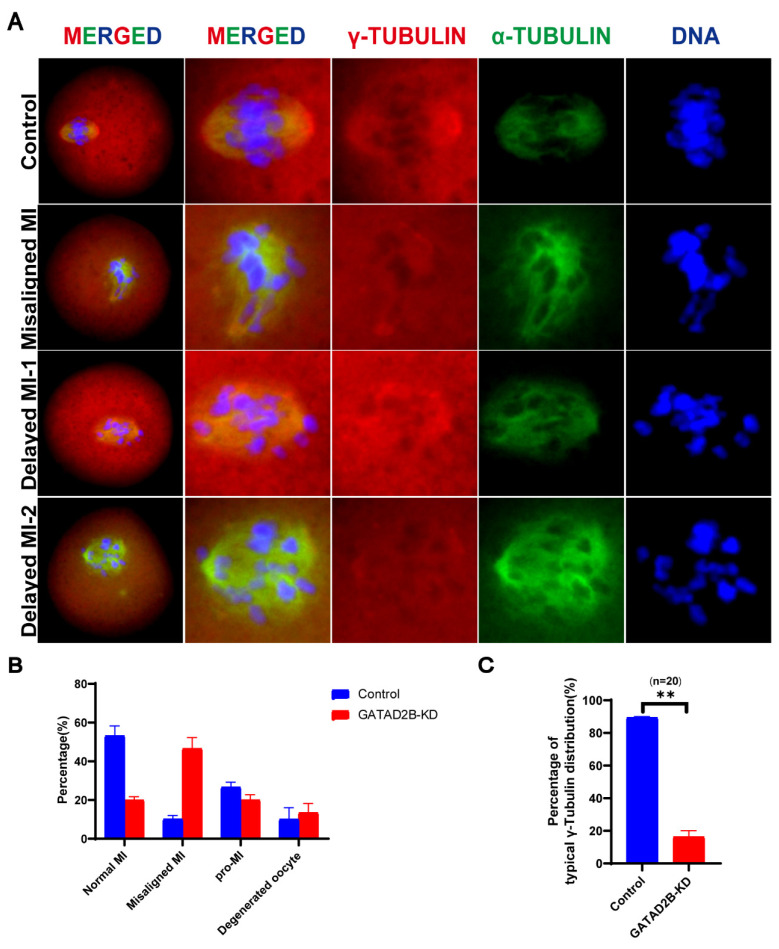
Knockdown of GATAD2B in oocytes leads to abnormal MTOC distribution. (A) Abnormal localization of γ-TUBB and α-TUBB in GATAD2B-KD oocytes. (B) Bar graph showing the quantitative analysis of developmental stages (Pro-MI, MI, Misaligned MI, etc.) during oocyte maturation for 9 hours. Data are presented as mean±SEM. (C) Bar graph representing the proportion of well-aligned γ-TUBB in GATAD2B-KD oocytes and control group oocytes. Scale bar, 20 μm. Spindle poles of more than 20 oocytes were analyzed. ** ρ<0.01. MI, metaphase I; MTOC, microtubule organizing center; SEM, standard error of the mean.

GATAD2B-KD oocytes exhibited maturation defect when cultured to MII stage. However, it remains unclear whether GATAD2B-KD affects microtubule and, accordingly, chromosome arrangement. When resuming meiosis, oocytes first form MTOC. Subsequently, microtubules, centered on the MTOC, extend rapidly. The MTOCs will be distributed at both ends of the spindle, leading the movement of spindle. With GATAD2B knockdown, disorganized enrichment of spindle pole was significantly observed, which suggested the presence of abnormalities in the MTOCs. In order to confirm the formation and function of MTOCs, one core constituent protein, γ-tubublin, of the MTOCs were stained. Notable is, γ-tubublin, ahorseshoe-like-distributed protein in normal MI oocytes, showed abnormal distribution in GATAD2B-KD oocytes, leading to abnormal microtubule attachment (Figures 7A, 7C). Therefore, it is reasonable that during the MI period, the morphology of the spindle labeled by α-tubulin has been abnormal in GATAD2B-KD oocytes. Similarly, chromosome arrangement is already disordered in GATAD2B-KD MI oocytes (Figure 7B). Specifically, after 9 hours of oocyte maturation, only about 20% of the GATAD2B-KD oocytes entered the typical MI stage and in more than 40% of GATAD2B-KD oocytes, the chromosomes were not neatly arranged. This suggests that GATAD2B-KD affects chromosome arrangement and oocyte maturation as early as MI stage.

### Knockdown of GATAD2B perturbs histone deacetylation

**Figure 8:**
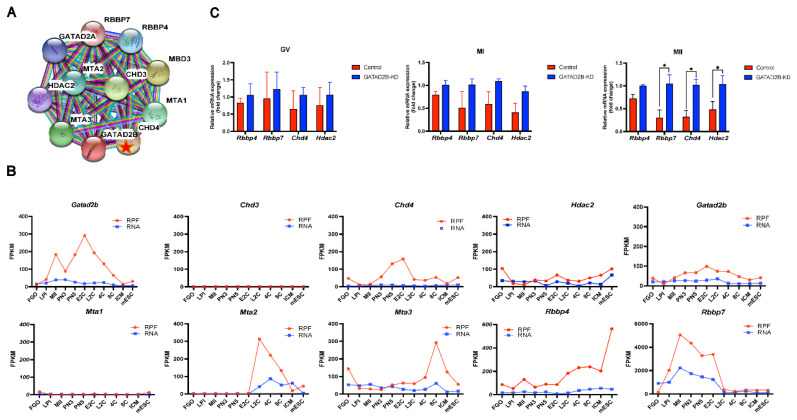
Changes in expression of GATAD2B and other major components of the NuRD complex. (A) PPI showing the proteins that GATAD2B may interact with in the NuRD complex. (B) Dynamic expression changes of each major component of the NuRD complex from the oocyte to the ICM. FGO, fully grown oocyte; LPI, late prometaphase I (6 h after meiotic resumption *in vitro*); MII, metaphase II; PN3, early one-cell stage; PN5, late one-cell stage; E2C, early two-cell stage; L2C, late two-cell stage; 4C, four-cell stage; 8C, eight-cell stage; ICM, inner cell mass of blastocyst stage; mESC, mouse embryonic stem cell. (C) Changes in the representative components of the NuRD complex at different times (GV, MI, MII) during GATAD2B-KD oocyte maturation. Datas are presented as mean±SEM. * ρ<0.05. GV, germinal vesicle; MI, metaphase I; MII, metaphase II; SEM, standard error of the mean.

To figure out the role of GATAD2B in oocyte maturation, fully grown oocytes with intact nucleus were injected with a mixture of siRNA oligonucleotide, which is necessary to effectively deplete GATAD2B’s RNA stores. This approach significantly suppressed the translation of GATAD2B protein associated with maturation (Figure 3). *Gatad2b* is a key component of the NuRD complex, and we sought to investigate whether *Gatad2b* knockdown would affect the expression of other components of the NuRD complex. Multiple components of the NuRD complex show different patterns of change during oocyte maturation and preimplantation embryonic development (Figures 8A, 8B). We detected the mRNA expression of main components, such as *Rbbp4*, *Rbbp7*, *Chd4*, and *Hdac2*, of NuRD complex. These NuRD complex components did not decrease significantly with knockdown of *Gatad2b* during GV and MI. However, the mRNA expression of *Rbbp7*, *Chd4*, and *Hdac2* was significantly increased in *Gatad2b*-KD oocytes during the MII period (Figure 8C).

**Figure 9:**
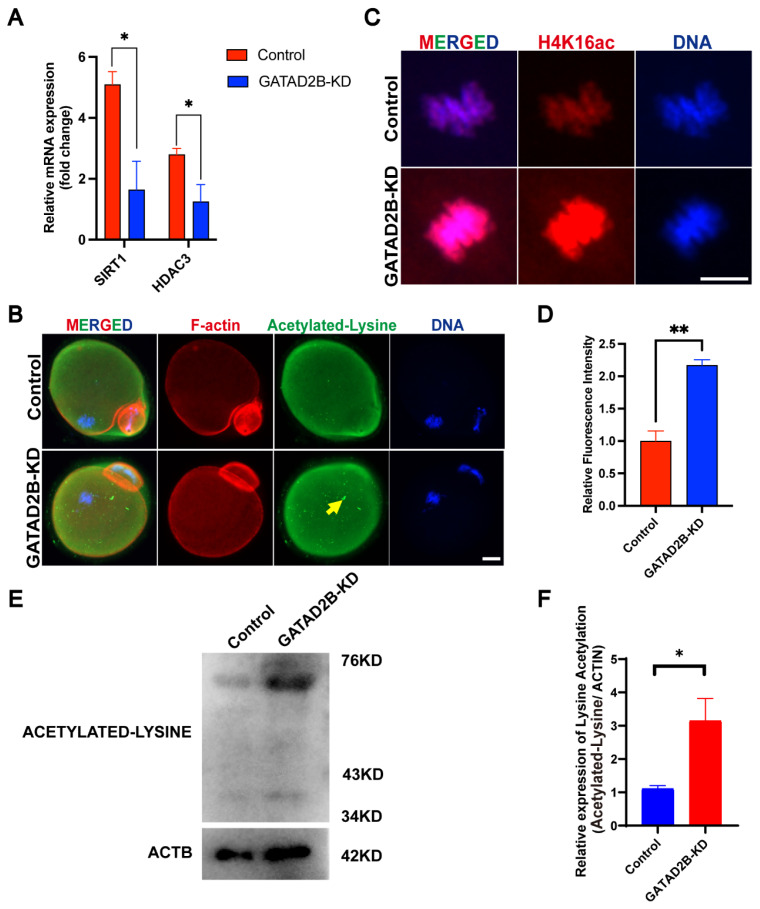
Increased protein acetylation in GATAD2B-KD oocytes. (A) Plot of changes in acetylation-related factors in GATAD2B-KD oocytes. Data are presented as mean±SEM. * ρ<0.05. (B) Immunofluorescence plot of oocyte protein lysine acetylation. Arrows indicate abnormal acetylation accumulation. Scale bar, 20 μm. (C) Immunofluorescence plot of representative histones of oocytes. Scale bar, 5 μm. (D) Statistical histogram of grayscale values of H4K16ac. Data are presented as mean±SEM. At least 20 oocytes were analyzed in each independent experiment. (E) Western blot analysis of protein lysine acetylation in GATAD2B-KD oocytes. (F) Bar graph displaying the grayscale values of protein lysine acetylation in GATAD2B-KD oocytes. SEM, standard error of the mean. * ρ<0.05, ** ρ<0.01.

*Gatad2b* is a key component of the NuRD complex, and *Gatad2b* knockdown may affect oocyte protein acetylation. The mRNA expressions of Multiple deacetylation-related factors, *Sirt1* and *Hdac3*, also decreased to varying degrees. Thereinto, deacetylase *Sirt1* decreased by 5 times nearly (Figure 9A). Next, to evaluate the role of GATAD2B in regulating protein deacetylation in oocytes, we examined GATAD2B-KD oocyte lysine acetylation by immunocytochemistry. Lysine acetylation in MII oocytes appears to be pervasive in the cytoplasm, but there is an abnormal accumulation of lysine acetylation in the cytoplasm of knockout oocytes (Figure 9B). Acetylation not only exists in cytoplasmic proteins, but also in intranuclear proteins. Deacetylation of H4K16 was significantly inhibited in GATAD2B-KD oocytes. Quantitative analysis showed that the acetylation level of H4K16 was significantly increased by nearly 2 times (Figures 9C, 9D). In addition, we reconfirmed by Western Blot that the total lysine acetylation level in GATAD2B-KD oocytes significantly increased compared with the control (Figures 9E, 9F). These results suggest that GATAD2B may regulate the process of protein deacetylation during oocyte maturation.

## DISCUSSION

Here, we show for the first time that *Gatad2b* can regulate lysine deacetylation of non-nuclear proteins and histone deacetylation of nuclear proteins H4K16 (Figure 9). In addition, *Gatad2b* expression was most abundant during the MII period of meiotic maturation (Figure 1). The specific localization of *Gatad2b* at each stage of meiotic maturation in oocytes was also described in detail (Figure 2). GATAD2B-KD oocytes exhibit delayed oocyte meiotic resumption, abnormal chromosome arrangement, abnormal spindle assembly, and increased aneuploidy (Figures 4, 5, 7). This phenotype is consistent with the deletion of RBBP4, RBBP7, and HDAC2 in oocytes [17,19,24]. RBBP7-mediated histone acetylation is required for localization and function of the CPC [17]. RBBP4 regulates bipolar spindle assembly by affecting AURKC function [19]. Unlike these two, we found that *Gatad2b* loss of function resulted in significant abnormalities in MTOC assembly (Figure 6).

**Figure 10:**
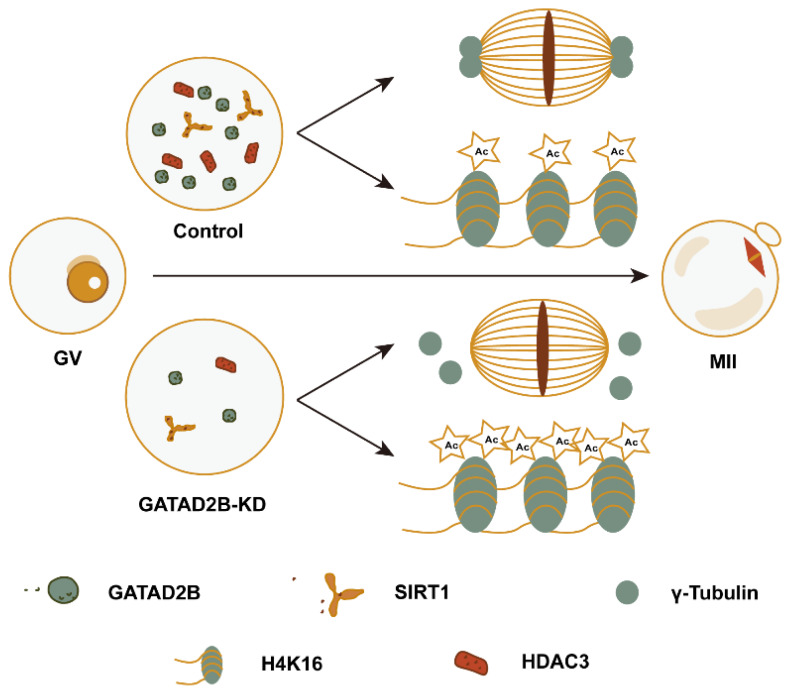
GATAD2B regulates histone deacetylation and spindle assembly pattern in oocytes. The expression of acetylation-related factors such as SIRT1 and HDAC3 were reduced in GATAD2B-KD oocytes. This resulted in abnormal distribution of MTOC core protein γ-TUBB, and abnormal accumulation of nuclear protein acetylation histone H4K16ac, cytoplasmic protein lysine acetylation. MTOC, microtubule organizing center.

In GATAD2B-KD oocytes, spindle poles were significantly narrowed (Figure 5). Spindle poles are enriched MTOC in oocytes, similar to mitotic centrosomes with attached microtubules. Numerous protein profiling databases in recent years suggest that in addition to chromosome-associated proteins such as histones, numerous components of centrosomes are also acetylated [25–28]. The acetylation of centrosomal proteins is widely distributed in eukaryotic cells. In mitosis, the centrosome protein PLK2 is acetylated and undergoes deacetylation by SIRT1. Acetylation protects PLK2 from ubiquitination, and SIRT1-mediated deacetylation promotes ubiquitin-dependent degradation of PLK2. SIRT1 controls centriole duplication by temporally modulating centrosomal PLK2 levels [29]. Centrosomal proteins such as pericentry and γ-tubulin, undergo acetylation and deacetylation during oocyte meiosis [30]. In oocyte meiosis, RBBP4 is one of the major components of the NuRD deacetylation complex. In RBBP4-depleted oocytes, MTOC migration processes into two spindle poles requires deacetylation of γ-tubulin because the number of γ-tubulin foci increased more [19]. Similarly, in GATAD2B-KD oocytes,γ-tubulin, which is major component of MTOC, do not form the typical horseshoe-like distribution but are diffused in the oocyte cytoplasm (Figures 6, 10).

During oocyte maturation, histone deacetylation is essential for accurate chromosome segregation that is required to generate healthy, developmentally competent oocytes [24]. *Gatad2b*, which is one of the NuRD complex components, can recruit *Mbd2*, another component of the NuRD complex, to DNA and histones and affect histone deacetylation and repression of transcription [13,31]. *Gatad2b* can also be sumoylated to enhance the formation of the NuRD complex [15,16]. In mouse ESC, Co-Immunoprecipitation showed that *Gatad2b* interacted with H3k9ac, H3K27ac and H4K16ac [14]. Insufficient histone deacetylation of H4K12 during meiosis in mouse oocytes is associated with chromosome misalignment and aneuploidy [32]. Inhibition of HDAC activity by trichostatin A (TSA) treatment resulted in severe chromosome misalignment, chromosome lag, abnormal cytoplasmic segregation, and abnormal chromosome segregation during mouse oocyte maturation [33]. In RBBP4 or RBBP7-depleted oocytes, histones H3K4, H4K8, H4K12, and H4K16 were hyperacetylated [17,19]. A similar result was found for a significant increase in histone H4K16ac in GATAD2B-KD oocytes. In addition to nuclear histone H4K16ac proteins, a large number of non-nuclear proteins were also hyperacetylated in GATAD2B-depleted oocytes (Figure 9) [7]. These results provide evidence that GATAD2B as a novel regulator of histone deacetylation during oocyte maturation.

The Oocyte-to-Embryo transition (OTE) process is initiated with the resumption of oocyte meiosis. This process involves the initiation of degradation and translational activation of numerous unstable maternal mRNAs stored in the cytoplasm [34,35]. Although translation and degradation of maternal mRNA occur primarily in mature oocytes, these mechanisms are essential for oocytes and zygotes to establish their ability to complete Maternal-to-zygotic transition (MZT) [36–39]. Interestingly, we observed that *Gatad2b* expression was highest during MII and was enriched in the first polar body (Figure 1). Further, *Gatad2b* maintained high expression in zygotes (Figure 8). This suggests that *Gatad2b* may have an important role in the OTE process. *Gatad2b* expression trend from oocytes to preimplantation embryos is different from other components of the deacetylation complex NuRD, and it can be speculated that *Gatad2b* has a unique function during early embryonic development. Therefore, the function of *Gatad2b* in the OTE process needs to be further explored.

## CONCLUSION

This study describes *Gatad2b* expression and localization during oocyte maturation. The absence of GATAD2B will lead to the abnormal distribution of the MTOC, the abnormal spindle assembly, chromosome misalignment, and the increase of aneuploidy. These results implicate GATAD2B as a novel regulator of histone deacetylation during oocyte maturation and provide evidence that such deacetylation is required for proper spindle assembly (Figure 10).

## References

[1] Mikwar M, MacFarlane AJ, Marchetti F (2020). Mechanisms of oocyte aneuploidy associated with advanced maternal age. Mutat Res Rev Mutat Res.

[2] Ma JY, Li S, Chen LN, Schatten H, Ou XH, Sun QY (2020). Why is oocyte aneuploidy increased with maternal aging?. J Genet Genomics.

[3] Huang JC, Yan LY, Lei ZL (2007). Changes in histone acetylation during postovulatory aging of mouse oocyte. Biol Reprod.

[4] Manosalva I, González A (2009). Aging alters histone H4 acetylation and CDC2A in mouse germinal vesicle stage oocytes. Biol Reprod.

[5] Huang R, Sui L, Fu C (2021). HDAC11 inhibition disrupts porcine oocyte meiosis via regulating α-tubulin acetylation and histone modifications. Aging.

[6] Samata M, Alexiadis A, Richard G (2020). Intergenerationally maintained histone H4 lysine 16 acetylation is instructive for future gene activation. Cell.

[7] Verdin E, Ott M (2015). 50 Years of protein acetylation: from gene regulation to epigenetics, metabolism and beyond. Nat Rev Mol Cell Biol.

[8] Kim JM, Liu H, Tazaki M, Nagata M, Aoki F (2003). Changes in histone acetylation during mouse oocyte meiosis. J Cell Biol.

[9] Wu X, Hu S, Wang L, Li Y, Yu H (2020). Dynamic changes of histone acetylation and methylation in bovine oocytes, zygotes, and preimplantation embryos. J Exp Zool B Mol Dev Evol.

[10] Wang H, Cai H, Wang X (2019). HDAC3 maintains oocyte meiosis arrest by repressing amphiregulin expression before the LH surge. Nat Commun.

[11] dos Santos RL, Tosti L, Radzisheuskaya A (2014). MBD3/NuRD facilitates induction of pluripotency in a context-dependent manner. Cell Stem Cell.

[12] Mor N, Rais Y, Sheban D (2018). Neutralizing Gatad2a-Chd4-Mbd3/NuRD complex facilitates deterministic induction of naive pluripotency. Cell Stem Cell.

[13] Brackertz M, Gong Z, Leers J, Renkawitz R (2006). p66α and p66β of the Mi-2/NuRD complex mediate MBD2 and histone interaction. Nucleic Acids Res.

[14] Xing G, Liu Z, Huang L (2022). MAP2K6 remodels chromatin and facilitates reprogramming by activating Gatad2b-phosphorylation dependent heterochromatin loosening. Cell Death Differ.

[15] Gong Z, Brackertz M, Renkawitz R (2006). SUMO modification enhances p66-mediated transcriptional repression of the Mi-2/NuRD complex. Mol Cell Biol.

[16] Torchy MP, Hamiche A, Klaholz BP (2015). Structure and function insights into the NuRD chromatin remodeling complex. Cell Mol Life Sci.

[17] Balboula AZ, Stein P, Schultz RM, Schindler K (2014). Knockdown of RBBP7 unveils a requirement of histone deacetylation for CPC function in mouse oocytes. Cell Cycle.

[18] Verreault A, Kaufman PD, Kobayashi R, Stillman B (1998). Nucleosomal DNA regulates the core-histone-binding subunit of the human Hat1 acetyltransferase. Curr Biol.

[19] Balboula AZ, Stein P, Schultz RM, Schindler K (2015). RBBP4 regulates histone deacetylation and bipolar spindle assembly during oocyte maturation in the mouse. Biol Reprod.

[20] Cao G, Li M, Wang H, Shi L, Su YQ (2018). Interference with the C-terminal structure of MARF1 causes defective oocyte meiotic division and female infertility in mice. J Biomed Res.

[21] Yao Q, Cao G, Li M (2018). Ribonuclease activity of MARF1 controls oocyte RNA homeostasis and genome integrity in mice. Proc Natl Acad Sci USA.

[22] Boroviak T, Stirparo GG, Dietmann S (2018). Single cell transcriptome analysis of human, marmoset and mouse embryos reveals common and divergent features of preimplantation development. Development.

[23] Xiong Z, Xu K, Lin Z (2022). Ultrasensitive Ribo-seq reveals translational landscapes during mammalian oocyte-to-embryo transition and pre-implantation development. Nat Cell Biol.

[24] Ma P, Schultz RM (2013). Histone deacetylase 2 (HDAC2) regulates chromosome segregation and kinetochore function via H4K16 deacetylation during oocyte maturation in mouse. PLOS Genet.

[25] Lundby A, Lage K, Weinert BT (2012). Proteomic analysis of lysine acetylation sites in rat tissues reveals organ specificity and subcellular patterns. Cell Rep.

[26] Weinert BT, Scholz C, Wagner SA (2013). Lysine succinylation is a frequently occurring modification in prokaryotes and eukaryotes and extensively overlaps with acetylation. Cell Rep.

[27] Chen Y, Zhao W, Yang JS (2012). Quantitative acetylome analysis reveals the roles of SIRT1 in regulating diverse substrates and cellular pathways. Mol Cell Proteomics.

[28] Fournier M, Orpinell M, Grauffel C (2016). KAT2A/KAT2B-targeted acetylome reveals a role for PLK4 acetylation in preventing centrosome amplification. Nat Commun.

[29] Ling H, Peng L, Wang J, Rahhal R, Seto E (2018). Histone deacetylase SIRT1 targets Plk2 to regulate centriole duplication. Cell Rep.

[30] Ling H, Peng L, Seto E, Fukasawa K (2012). Suppression of centrosome duplication and amplification by deacetylases. Cell Cycle.

[31] Feng Q, Cao R, Xia L, Erdjument-Bromage H, Tempst P, Zhang Y (2002). Identification and functional characterization of the p66/p68 components of the MeCP1 complex. Mol Cell Biol.

[32] Akiyama T, Nagata M, Aoki F (2006). Inadequate histone deacetylation during oocyte meiosis causes aneuploidy and embryo death in mice. Proc Natl Acad Sci USA.

[33] Yang F, Baumann C, Viveiros MM, De La Fuente R (2012). Histone hyperacetylation during meiosis interferes with large-scale chromatin remodeling, axial chromatid condensation and sister chromatid separation in the mammalian oocyte. Int J Dev Biol.

[34] Sha QQ, Zhang J, Fan HY (2019). A story of birth and death: mRNA translation and clearance at the onset of maternal-to-zygotic transition in mammals. Biol Reprod.

[35] Svoboda P, Franke V, Schultz RM (2015). Sculpting the transcriptome during the oocyte-to-embryo transition in mouse. Curr Top Dev Biol.

[36] Tadros W, Lipshitz HD (2009). The maternal-to-zygotic transition: a play in two acts. Development.

[37] Chen J, Melton C, Suh N (2011). Genome-wide analysis of translation reveals a critical role for deleted in azoospermia-like (Dazl) at the oocyte-to-zygote transition. Genes Dev.

[38] Sha QQ, Yu JL, Guo JX (2018). CNOT6L couples the selective degradation of maternal transcripts to meiotic cell cycle progression in mouse oocyte. EMBO J.

[39] Yu C, Ji SY, Sha QQ (2016). BTG4 is a meiotic cell cycle–coupled maternal-zygotic-transition licensing factor in oocytes. Nat Struct Mol Biol.

